# Isolation and Use of *Coprothermobacter* spp. to Improve Anaerobic Thermophilic Digestion of Grass

**DOI:** 10.3390/molecules27144338

**Published:** 2022-07-06

**Authors:** Przemysław Liczbiński, Sebastian Borowski, Adriana Nowak

**Affiliations:** Department of Environmental Biotechnology, Faculty of Biotechnology and Food Science, Lodz University of Technology, Wolczanska 171/173, 90-530 Lodz, Poland; sebastian.borowski@p.lodz.pl (S.B.); adriana.nowak@p.lodz.pl (A.N.)

**Keywords:** *Coprothermobacter*, anaerobic digestion, hyperthermophilic pretreatment

## Abstract

The isolation of microorganisms was performed from digestate from the process of the anaerobic digestion (AD) of grass after hyperthermophilic pretreatment. The bacterium that was isolated and identified was *Coprothermobacter* sp. Using the isolated bacteria, an AD process on fresh grass (GB) and pretreated grass (PGB) was carried out with 10% of its addition. The highest methane yield of 219 NmlCH_4_/gVS was recorded for PGB at 55 °C. In contrast, fresh grass subjected to thermophilic digestion produced only 63 NmlCH_4_/gVS. Due to the addition of bacteria in the AD process, an increase in the efficiency of hydrogen and methane production was observed in both fresh grass and grass after pretreatment.

## 1. Introduction

Renewable energy sources are attracting more and more attention around the world, and their pace of development is not slowing down. The increase in the world’s population and the use of fossil fuels will result in the need to obtain alternative energy sources that can reduce the growing emissions of greenhouse gases [1,2]. Anaerobic digestion (AD) is a biological process that converts organic waste into nutrient-rich digestate and biogas rich in methane and hydrogen, with high calorific value. The application of thermophilic conditions in AD improves the decomposition rate of organic matter, increases the methane yield, and allows for more efficient pathogen inactivation [3]. Anaerobic digestion, as a multi-stage process, consists of hydrolysis, acidogenesis, acetogenesis, and methanogenesis phases, each of which is characterized by a different microbiota, which creates a diverse microbial community. Hydrolysis is considered as a rate-limiting step for the whole digestion process. At this stage, complex organic polymers including proteins, lipids, and polysaccharides are degraded into simple sugars, fatty acids, amino acids, alcohols, and aldehydes [4,5]. The identification of the microorganisms involved in the hydrolysis stage and their use in AD may significantly increase the efficiency of the digestion process and biogas yield. In this context, bacteria from the genus *Coprothermbacter* are of great interest, especially when the process is performed at increased temperatures. In fact, for the first time, the genus *Coprothermboacter proteolyticus* was recently identified in anaerobic thermophilic digesters [6]. *Coprothermobacter* spp. is generally known for its proteolytic properties, and can therefore facilitate the processing of protein-rich waste at higher temperatures [7]. These bacteria were identified in anaerobic thermophilic processes treating sewage sludge, slaughterhouse waste, food scraps, and even cattle manure [8,9,10,11,12,13,14]. Moreover, the growth of these microorganisms in the protein-rich environment is associated with the production of hydrogen and methane, thanks to syntrophic relationships with hydrogenotrophic methanogenic archaea [14,15,16]. Hence, the aim of this study was the isolation and propagation, as well as the macro- and microscopic and genetic identification, of the isolated microorganisms from a thermophilic anaerobic digester operated with grass. Additionally, the use of isolated microorganisms to increase the efficiency of biogas production under thermophilic conditions was also evaluated. To the best of the authors’ knowledge, this is the first study in which *Coprothermobacter* spp. was isolated from a thermophilic AD reactor treating grass and then used to increase the efficiency of biogas production for the same substrate and process conditions. According to the authors’ knowledge, this is the first study in which the *Coprothermobacter* spp. bacteria were used to increase the anaerobic digestion and methane and hydrogen yield from grass.

## 2. Results and Discussion

**Figure 1:**
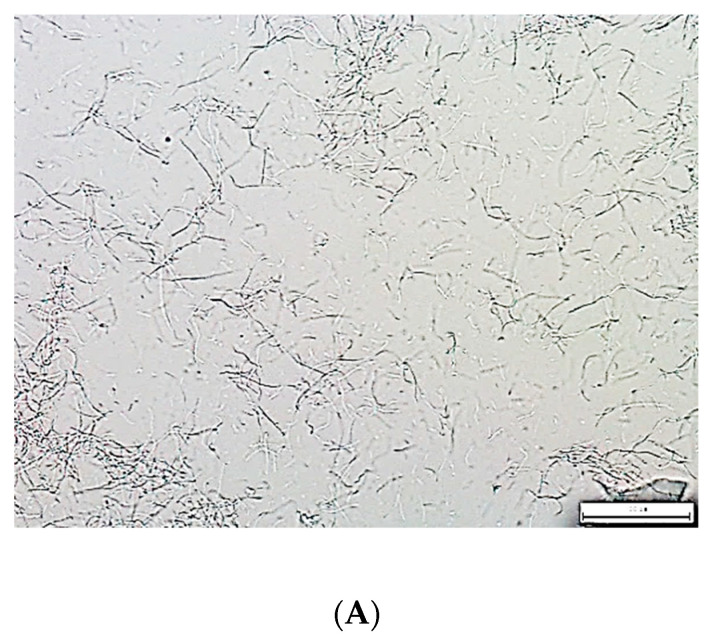

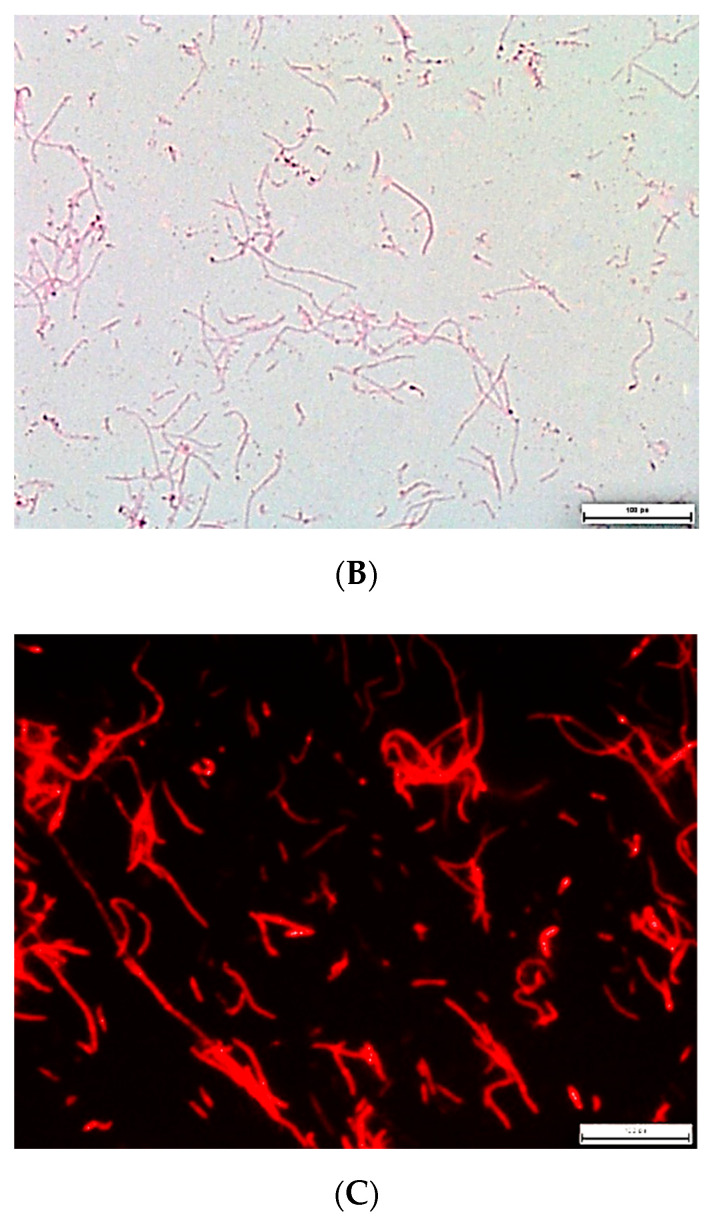
Microphotographs of *Coprothermobacter* spp. isolate: (**A**) direct preparation (40× magnification), (**B**) Gram staining (100× magnification), (**C**) propidium iodide staining (100× magnification) (Nikon Eclipse Ci H600 L, Tokyo, Japan).

Metagenomic analysis revealed 99.73% of bacteria from the *Coprothermobacteraceae* family. *Coprothermobacter* spp. accounted for 99.6% and *Coprothermobacter proteolyticus* accounted for 0.13%. Isolated *Coprothermobacter* sp. bacteria were Gram-negative, rod-shaped, anaerobic, and thermophilic, detected mainly at high temperatures (from 50 to 70 °C) and in strictly anaerobic conditions, which justifies their presence in the process of grass AD at 55 °C. The morphology of the isolate is presented in Figure 1. In addition, it was found that bacteria of this genus show a strong ability to degrade proteins and peptides, and are also important producers of hydrogen [14]. Moreover, *Coprothermobacter* spp. has also been identified in the thermophilic AD of sewage sludge [17]. Similar results were obtained when comparing the mesophilic and thermophilic communities of microorganisms during the AD of food waste. Similarly, under thermophilic conditions, the presence of *Coprothermobacter* spp. was observed [18]. Interestingly, *Coprothermobacter* spp. was also identified in AD substrates such as lawn clippings and branches and wood clippings. In addition, cellulose degradation ability for these bacteria was observed [19]. These studied substrates were almost the same as the ones that we used in the current research.

### Anaerobic Digestion Process

**Figure 2:**
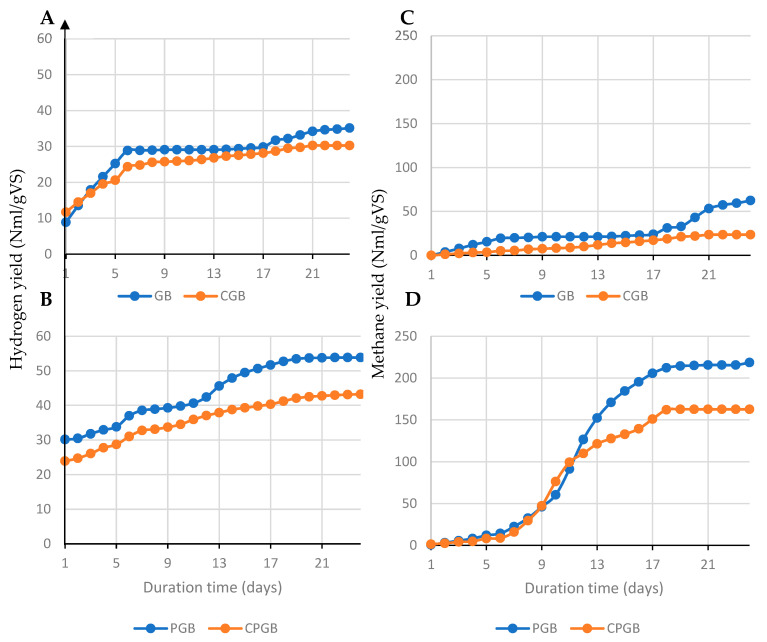
Cumulative hydrogen and methane production for AD pretreated grass with bacteria with control sample (PGB and CPGB, respectively: **B**,**C**) and raw grass with bacteria with control sample (GB and CGB, respectively: **A**,**D**) at 55 °C.

The hydrogen and methane yields reported from the experiments are plotted in Figure 2, whereas the characteristics of the digestates from the experiments are summarized in Table 1. As shown in Figure 2, the addition of the *Coprothermobacter* spp. culture resulted in a significant increase (*p* < 0.05) in hydrogen production for both pretreated and untreated grass. However, much higher hydrogen yield was observed in pretreated grass (PGB) (54 NmLH2/gVS), compared to the production observed in fresh grass (GB) (35 NmLH2/gVS). This indicates the highest efficiency of the combined thermal and biological pretreatment, mostly improving the hydrogen yield and, hence, the hydrolysis rate of grass. It is hypothesized that thermal pretreatment loosens the lignocellulosic structure of grass to make it more available for bacteria of *Coprothermobacter* spp., which provide further decomposition of organic polymers. The addition of *Coprothermobacter* spp. also increased the production of methane (*p* < 0.05), and the results corresponded to the yields of hydrogen. Hence, the highest methane yield of 219 NmlCH_4_/gVS, was reported in the experiment with hyperthermophilically pretreated grass subjected to digestion with the addition of the *Coprothermobacter* spp. culture. In contrast, the AD process of grass with neither thermal pretreatment nor the addition of *Coprothermobacter* spp. gave only 62 NmlCH_4_/gVS. The analysis of the digestates revealed increased values of both TVFA and nutrients in the experiments with *Coprothermobacter* spp. addition; however, the differences with the control were not high. In detail, the addition of the microbial culture to grass increased the amount of ammonium nitrogen by 32% for the GB variant and by 11% for the PGB variant. Furthermore, an increase in TVFA from 8800 to 9110 mg/L was observed for the pretreated grass and from 5169 to 6150 mg/L for the untreated grass. The changes in phosphates were negligible, whereas the pH slightly dropped with the addition of *Coprothermobacter* spp. The increase in the values of TVFA, ammonium nitrogen, and orthophosphates may indicate a greater degree of hydrolysis.

The high efficiency of hydrolysis and the increase in biogas production in the tested variants with the addition of *Coprothermobacter* spp. may be related to the content and availability of proteins. This relationship was also observed during the thermophilic AD of activated sludge [13]. In addition, *Coprothermobacter* strains have been detected during the anaerobic digestion of various protein-rich wastes [16,20,21]. The increased level of hydrolysis in this research could be linked to the degradation of proteins by *Coprothermobacter* spp. as these bacteria follow amino acid fermentation [11]. Moreover, *Coprothermobacter* spp. is involved in the degradation of low-molecular-weight organic compounds, which may have been released after the thermal pretreatment, and this phenomenon was also observed during the AD of solid organic waste [21]. In turn, the greater methane production can be explained by the fact that *Coprothermobacter* spp., by producing hydrogen, can promote syntrophic cooperation with methanogens. Hydrogen is one of the carriers in interspecies electron transfer [14]. *Coprothermobacter* spp. can degrade proteins in syntrophic association with methanogens, and hence, by forming a syntrophy with methanogens, the activity of *Coprothermobacter* spp. is improved. Moreover, hydrogen is the main source of energy for *Methanothermobacter thermoautotrophicus*, often identified in thermophilic anaerobic digestion systems [16,22].

## 3. Materials and Methods

### 3.1. Material

Anaerobic digestion experiments were performed using grass from a home garden, which was collected in October 2021. After harvesting, the raw, wet grass was ground in a shredder (FIMAR TS-32D400V) in order to obtain a size reduction to 3–5 mm, and then stored at −18 °C. The composition of the grass was as follows: carbon—58.7% TS; nitrogen—2.6%TS; phosphorus—0.8% TS.

### 3.2. Isolation of Thermophilic Bacteria

**Figure 3:**
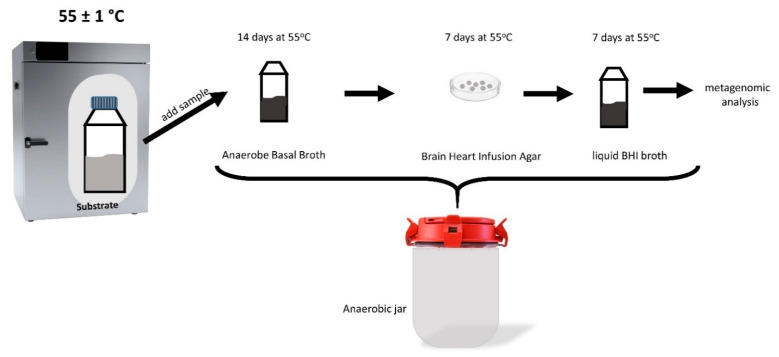
Laboratory stage of microbial isolation.

The material used for the isolation of microorganisms was taken from the digestate from the AD process, which was carried out at a temperature of 55 °C, in which the substrate was grass subjected to an earlier hyperthermophilic treatment, where the maximum efficiency of both hydrogen and methane production was 41 mLH_2_/gVS and 160 mLCH_4_/gVS, respectively. Anaerobe Basal Broth (Oxoid) was inoculated with 10% of the digestate and the microorganisms were multiplied for 14 days at 55 °C in anaerobic conditions. Next, streak plating was performed on Brain Heart Infusion Agar (BHI, Merck Life Science Poznań, Poland) and samples were incubated for 7 days at 55 °C in an anaerobic jar with the addition of an AnaeroGen^TM^ Armosphere Generation Systems sachet (Thermo Scientific, Waltham, MA, USA) (Figure 3). After the isolation of single colonies, each colony of a different morphology (in color, shape, or surface), as assessed macroscopically, was transferred to the liquid BHI broth and multiplied for 7 days at 55 °C in anaerobic conditions. Among the few isolates on the petri dish, only 1 was able to multiply. This isolate was genetically identified.

#### 3.2.1. Genetic Identification

The isolated pure bacterial culture was subjected to metagenomic analysis (Figure 1). Prior to the metagenomic analysis of the bacterial culture, the sample was stored at −20 °C. The metagenomic analysis of the bacterial population was performed on the basis of the hypervariable V3–V4 region of the 16S rRNA gene. Sequences of the 341F and 785R primers were used to prepare the library. PCR was carried out using a Q5 Hot Start High-Fidelity 2× Master Mix. Sequencing was performed on a MiSeq apparatus, in paired-end technology, 2 × 300 nt, using the Illumina v3 kit. Bioinformatic analysis was performed with QIIME 2 based on the Silva 138 reference sequence and DADA2 package.

#### 3.2.2. Microscopic Observations and Staining

Microscopic photographs of the direct preparation and the preparations stained with the Gram method and fluorescent propidium iodide were taken. After air-drying and fixing, the cells were stained with 1 µg/mL propidium iodide in the dark. The morphology of cells was observed at 40× or 100× objective under a fluorescent/contrast-phase microscope (Nikon Eclipse Ci H600L, Tokyo, Japan) attached to a digital camera (Nikon Digital Sight DS-U3, Tokyo, Japan) and using imaging software (NIS-elements BR 3.0, Nikon).

### 3.3. Anaerobic Digestion Experiments 

The isolated and identified strain of *Coprothermobacter* spp. was cultivated and multiplied as a pure culture on liquid BHI broth, in strictly anaerobic conditions, in seal-cup tubes at the temperature of 55 °C and with the addition of an AnaeroGenTM Systems sachet.

The AD process with the addition of isolated bacteria was carried out in a similar manner to the process carried out in previous studies [23]. The AD was carried out in two variants: I—grass pretreated at 70 °C, mixed with water in the ratio 1:2, with the addition of bacteria (PGB); and II—raw grass mixed with water in the ratio 1:2, with the addition of bacteria (GB). For each of the tested variants, a control system in which no bacteria were added was used, named CGB and CPGB, respectively, for GP and PGB. For the inoculation of the fermentation mixtures, cultures with the volume of 70 mL (6 × 10^8^ CFU/mL, i.e., 2.0 according to McFarland standard) were used, which constituted 10% of the volume (1:9 *v*/*v*). Before the bacteria were inoculated into the fermentation mixture, the bacterial cultures were centrifuged. Biomass containing no substrate was added to the fermentation mixture. The AD process was carried out in bottles with a total and working volume of 1 and 0.7 dm^3^, respectively.

### 3.4. Chemical Analyses

Ammonium nitrogen was analyzed and total volatile fatty acids (TVFA) determined by HACH-Lange test no. LCK 514, 8038, and LCK365 using the DR3900 spectrophotometer. Moreover, the analysis of orthophosphates (PO_4_^−3^) was performed via the HACH-Lange method no. 8048. Tests were performed according to the manufacturer’s instructions. The analysis of the produced biogas was performed using the GA−21plus gas analyzer (Madur, Zgierz, Poland). All the conducted research was performed in three repeats. Data processing and average value calculations were performed in Microsoft Excel 2010.

### 3.5. Statistical Analysis

Data obtained were subjected to statistical evaluation using one-way ANOVA analysis with OriginPro 6.1 (Northampton, MA, USA) software, at the significance level of *p* < 0.05.

## 4. Conclusions

The study showed that the isolated and identified microorganism of *Coprothermobacter* spp. can successfully improve the biogas yield from both fresh grass and grass after hyperthermophilic pretreatment. The anaerobic digestion of hyperthermophilically pretreated grass was found to be the most efficient as 54 NmLH_2_/gVS and 219 NmLCH_4_/gVS of hydrogen and methane were yielded, in contrast to the corresponding values reported for raw grass (35 NmLH_2_/gVS and 63 NmLCH_4_/gVS). The greatest improvement in both hydrogen and methane production can be achieved by combining thermal pretreatment with the addition of biological amendments to grass before being subjected to anaerobic digestion.

## Figures and Tables

**Table 1:** Concentrations of TVFA, ammonium nitrogen, orthophosphates (PO4−3), and pH in the digestates from the experiments. PGB—pretreated grass with bacteria; CPGB—control sample for PGB; GB—raw grass with bacteria; CGB—control sample for GB.

Name	GB	CGB	PGB	CPGB
Substrate	Grass + Bacteria	Control	Pretreated Grass + Bacteria	Control
Ammonium nitrogen (mg/L)	540 ± 41.9	408 ± 21.5	472 ± 14.4	423 ± 33.0
TVFA (mg/L)	9110 ± 548.5	8800 ± 565.7	6150 ± 393.7	5160 ± 224.8
Phosphorus (mg/L)	482 ± 26.8	476 ± 12.1	256 ± 41.7	240 ± 32.7
pH	7.4 ± 0.4	7.2 ± 0.6	7.4 ± 0.5	7.38 ± 0.3
Cumulative hydrogen yield (NmL/gVS)	35.1 ± 1.4	30.3 ± 0.4	54.1 ± 6.3	43.2 ± 0.6
Cumulative methane yield (NmL/gVS)	62.5 ± 2.7	23.6 ± 0.8	218.6 ± 5.1	162.6 ± 2.4

± standard deviation.

## Data Availability

The data presented in this study are available in this article and are available from the corresponding author upon reasonable request.

## Sample Availability

Samples of the compounds are not available from the authors.
